# Clinical research updates

**DOI:** 10.1111/camh.70084

**Published:** 2026-03-12

**Authors:** Marinos Kyriakopoulos, Dimitrios Apostolou, Sofia Kourtidou, Alexandra Karakotia

**Affiliations:** ^1^ National and Kapodistrian University of Athens Athens Greece; ^2^ Department of Child and Adolescent Psychiatry Institute of Psychiatry Psychology and Neuroscience, King's College London London UK; ^3^ South London and Maudsley NHS Foundation Trust London UK

## Prenatal maternal depression and child behavioural and developmental outcomes

Dimitrios Apostolou

National and Kapodistrian University of Athens

Depression during pregnancy is a highly prevalent condition, affecting approximately one in five pregnant women, and has been consistently linked to adverse cognitive, emotional, and behavioural outcomes in offspring. Previous meta‐analyses have demonstrated associations with increased internalising and externalising problems, as well as elevated risk for attention‐deficit hyperactivity disorder (ADHD) and autism spectrum disorder (ASD) symptoms. However, prior syntheses relied primarily on aggregated published estimates rather than harmonised individual‐level data, limiting the ability to uniformly control for confounders, examine timing of exposure (prepregnancy, prenatal, postnatal), assess cumulative effects, or clarify inconsistent findings regarding sex differences.

Hermans et al. (2026) conducted the largest individual participant data (IPD) meta‐analysis to date, harmonising data from seven major European birth cohorts: Amsterdam Born Children and their Development (ABCD), Avon Longitudinal Study of Parents and Children (ALSPAC), Danish National Birth Cohort (DNBC), Étude des Déterminants pré et postnatals du développement et de la santé de l'Enfant (EDEN), Generation R Study, Nascita e INFanzia: gli Effetti dell'Ambiente (NINFEA), and Prediction and prevention of preeclampsia and intrauterine growth restriction study (PREDO). The total sample comprised 76,514 children with complete exposure, covariate, and at least one outcome measure. Eight developmental outcomes were examined across multiple domains, including internalising, externalising, ADHD, ASD, language, motor skills, and verbal intelligence. The analyses further investigated the role of prepregnancy and postnatal maternal depression, cumulative exposure, and sex differences.

Results showed that children exposed to prenatal maternal depression scored on average 6–10 percentile points higher in internalising, externalising, ADHD, and ASD symptoms compared to unexposed children. These associations remained after adjusting for prepregnancy depression, underscoring the prenatal period as a critical window of vulnerability, potentially reflecting intrauterine mechanisms. Postnatal depression partially mediated these associations, and cumulative exposure across time points was associated with greater risk. No consistent associations were observed with motor skills, language, or verbal intelligence in the primary analyses, although continuous measures of depressive symptoms showed broader associations. Importantly, associations were comparable for male and female offspring.

Limitations of the study include potential shared‐rater bias, inability to fully address selection bias due to multicohort harmonisation constraints, partial overlap between ADHD and externalising measures in some cohorts, limited ASD data availability, and restriction to European populations.

In conclusion, this large‐scale harmonised IPD meta‐analysis provides robust evidence that prenatal maternal depression constitutes a significant risk factor for a broad range of offspring mental health symptoms. The prenatal period emerges as a sensitive window for intervention, highlighting the importance of early detection and preventive strategies targeting maternal depression during pregnancy to promote healthier developmental trajectories in children.

Hermans, A. P. C., Avraam, D., Schuurmans, I. K., Soares, A. G., Lahti‐Pulkkinen, M., Girchenko, P., … & El Marroun, H. (2026). Prenatal maternal depression and child behavioural and developmental outcomes: An individual participant data meta‐analysis in 76,514 children from the EU Child Cohort Network. *The Lancet Regional Health – Europe*, 63, 101595. https://doi.org/10.1016/j.lanepe.2026.101595

## Executive function predicts academic and social skills in autistic kindergartners

Sofia Kourtidou

National and Kapodistrian University of Athens

Autism spectrum disorder (ASD) is characterized by differences in social communication and restricted or repetitive behaviors and is frequently associated with executive function (EF) impairments, including weaknesses in working memory, inhibitory control, and cognitive flexibility. Meta‐analytic evidence indicates that EF difficulties emerge early in development and persist across the lifespan in autistic individuals. Although many autistic children demonstrate average intellectual ability, executive weaknesses may contribute substantially to variability in academic achievement and social adaptation. The transition from preschool to formal schooling represents a critical developmental period during which EF skills rapidly mature and increasingly support learning, classroom engagement, and peer interaction. However, prior research has largely relied on cross‐sectional designs or single‐method assessments, limiting understanding of how distinct aspects of EF longitudinally relate to functional outcomes.

Choi et al. (2025) used a longitudinal study design to address this gap by examining whether EF predicts academic (reading and mathematics) and social outcomes in autistic kindergarteners across the transition to formal schooling. The sample included 67 verbally fluent, cognitively able autistic children aged 4–6 years recruited from clinical and community settings. Diagnoses were confirmed using standardized procedures consistent with DSM‐5 criteria. Children were assessed at kindergarten entry and followed through the end of the school year. They were evaluated using a multimethod approach to capture complementary dimensions of EF. Performance‐based EF was assessed through the computerized EF Touch battery, which measures cognitive control and working memory. Behavioral self‐regulation was measured using the Head‐Toes‐Knees‐Shoulders task, which assesses a child's ability to follow complex instructions and control behavior in a structured setting. Everyday executive difficulties were evaluated via parent report using the BRIEF‐II Global Executive Composite, reflecting real‐life challenges in EF. Academic achievement was measured using standardized reading and mathematics assessments, and peer functioning was evaluated through parent‐reported peer play behaviors. Analyses controlled for age, sex, and nonverbal IQ to estimate the unique contribution of EF to outcomes.

Results indicated substantial variability in academic and social functioning at school entry. Performance‐based EF skills were significantly associated with concurrent academic performance and longitudinally predicted later mathematics achievement, even after accounting for demographic and cognitive factors. Observational EF measures also contributed to end‐of‐year mathematics outcomes. In contrast, parent‐reported executive difficulties were consistently associated with poorer peer play functioning, including greater social withdrawal and disruptive interactions, both concurrently and longitudinally. These findings indicate that performance‐based and observational EF primarily predict academic outcomes, whereas parent‐reported EF is more sensitive to everyday social functioning challenges. Importantly, performance‐based EF measures were not significantly related to social outcomes, underscoring distinct predictive patterns across assessment methods.

Limitations of the study include the evaluation of only verbal, predominantly male, cognitively able children which affects the generalizability of its findings; the lack of comparison with typically developing children; limited data on ethnicity; and some limitations related to measurements of EF.

Overall, the findings suggest that EF represents a foundational mechanism supporting early academic and social development in autistic children during the transition to formal schooling. Early, multidimensional assessment of EF may help educators and clinicians identify children at risk for academic and peer‐related difficulties and guide targeted, school‐based interventions, ensuring support is tailored to the child's specific EF profile.

Choi, B., Lee, H., Kuhn, L., Kim, J., Hong, S.J., Di Martino, A., … & Kim, S.H. (2026). Executive function predicts academic and social skills in autistic kindergartners based on a multimodal approach. *Journal of Child Psychology and Psychiatry*, 67(2), 225–237. doi: 10.1111/jcpp.70038.

## Benefits and harms of ADHD interventions: umbrella review and platform for shared decision making

Alexandra Karakotia

National and Kapodistrian University of Athens

Attention‐Deficit/Hyperactivity Disorder (ADHD) affects individuals in both childhood and adulthood. Despite the availability of various pharmacological and nonpharmacological interventions, certain studies have conflicting findings while the quality of evidence from systematic reviews and meta‐analyses remains inconsistent, leading to uncertainty for both clinicians and patients regarding effective treatment options.

Gosling et al. (2025) conducted an umbrella review of systematic reviews and meta‐analyses of randomized controlled trials comparing ADHD interventions with passive controls to assess treatment interventions for ADHD across the life span. They also developed a user‐friendly online platform to enhance accessibility to these findings. In total, 115 meta‐analytic reports were identified from six databases, searched through January 2025. Eligible meta‐analyses were reestimated, leading to 221 unique comparisons of treatment interventions. The primary outcomes assessed were ADHD symptoms severity, acceptability (all‐cause dropout), and tolerability (dropout due to side effects). The certainty of evidence for each outcome was assessed using an adaptation of the GRADE framework.

Regarding ADHD symptoms in children and adolescents, methylphenidate and amphetamines showed consistent benefits with moderate or high certainty of evidence. Nonstimulant medications such as atomoxetine, alpha‐2 agonists, and viloxazine also demonstrated efficacy, though the certainty of evidence was lower compared to stimulants. Preschoolers appeared to have similar results for methylphenidate. In adults, methylphenidate, atomoxetine, amphetamines (when only high‐quality trials were included), and only cognitive behavioral therapy among the nondrug interventions showed at least medium efficacy on ADHD symptoms, with moderate to high certainty of evidence. In children and adolescents, amphetamines showed worse tolerability than placebo, whereas methylphenidate appeared to have better acceptability. In adults, medications with demonstrated efficacy such as methylphenidate and atomoxetine tended to have worse tolerability than placebo. Regarding the secondary outcomes of this study, in children and adolescents, amphetamines were associated with improvement in academic performance and atomoxetine showed benefits in quality of life. In adults, atomoxetine improved emotional dysregulation, while methylphenidate was linked to better executive functioning.

The authors incorporate several methodological strengths in this study to support the robustness of its results, although they acknowledge several limitations related to the quality and scope of the existing meta‐analyses, as well as the inability to account for individual patient differences or combined treatment approaches. Overall, this umbrella review offers a comprehensive overview of evidence on ADHD treatments. The accompanying open‐access continuously updated online platform (https://ebiadhd‐database.org/) supports the accessibility to evidence‐based findings. Future research is recommended to assess the long‐term outcomes of ADHD interventions.

Gosling, C.J., Garcia‐Argibay, M., De Prisco, M., Arrondo, G., Ayrolles, A., Antoun, S., … & Cortese, S. (2025). Benefits and harms of ADHD interventions: umbrella review and platform for shared decision making. *British Medical Journal*, 391:e085875. doi: 10.1136/bmj‐2025‐085875.

## Funding information

No funders declared.

## Conflict of interest statement

M.K. is the *CAMH* Associate Editor for Clinical Research Updates. The editor thanks the contributors for this issue's Clinical Research Updates. The editor has declared that he has no competing or potential conflicts of interest in relation to this article, and all remaining authors declare no competing or potential conflict of interest.

## Ethics statement

No ethical approval was required for these updates.

## Data Availability

Data sharing is not applicable to this article as no new data were created or analyzed for these updates.

